# Changes in diagnosed diabetes, obesity, and physical inactivity prevalence in US counties, 2004-2012

**DOI:** 10.1371/journal.pone.0173428

**Published:** 2017-03-07

**Authors:** Linda S. Geiss, Karen Kirtland, Ji Lin, Sundar Shrestha, Ted Thompson, Ann Albright, Edward W. Gregg

**Affiliations:** 1 Centers for Disease Control and Prevention, Division of Diabetes Translation, Atlanta, Georgia, United States of America; 2 Northrop Grumman Corporation, Atlanta, Georgia, United States of America; Medical University Innsbruck, AUSTRIA

## Abstract

Recent studies suggest that prevalence of diagnosed diabetes in the United States reached a plateau or slowed around 2008, and that this change coincided with obesity plateaus and increases in physical activity. However, national estimates can obscure important variations in geographic subgroups. We examine whether a slowing or leveling off in diagnosed diabetes, obesity, and leisure time physical inactivity prevalence is also evident across the 3143 counties of the United States. We used publicly available county estimates of the age-adjusted prevalence of diagnosed diabetes, obesity, and leisure-time physical inactivity, which were generated by the Centers for Disease Control and Prevention (CDC). Using a Bayesian multilevel regression that included random effects by county and year and applied cubic splines to smooth these estimates over time, we estimated the average annual percentage point change (APPC) from 2004 to 2008 and from 2008 to 2012 for diabetes, obesity, and physical inactivity prevalence in each county. Compared to 2004–2008, the median APPCs for diabetes, obesity, and physical inactivity were lower in 2008–2012 (diabetes APPC difference = 0.16, 95%CI 0.14, 0.18; obesity APPC difference = 0.65, 95%CI 0.59, 0.70; physical inactivity APPC difference = 0.43, 95%CI 0.37, 0.48). APPCs and APPC differences between time periods varied among counties and U.S. regions. Despite improvements, levels of these risk factors remained high with most counties merely slowing rather than reversing, which suggests that all counties would likely benefit from reductions in these risk factors. The diversity of trajectories in the prevalence of these risk factors across counties underscores the continued need to identify high risk areas and populations for preventive interventions. Awareness of how these factors are changing might assist local policy makers in targeting and tracking the impact of efforts to reduce diabetes, obesity and physical inactivity.

## Introduction

Recent analyses of nationally representative data from two major population surveys suggest that the prevalence and incidence of diagnosed diabetes in the United States plateaued around 2008 [1, 2] and that incidence subsequently declined [3]. These changes occurred about the same time as improvements in several major diabetes risk factors, including a leveling off in obesity [4, 5], increases in physical activity [6–8], and improvements in diet [9–13]. However, national estimates of diabetes and its risk factors can obscure important variations in demographic, socioeconomic, health status, or geographic subgroups. Type 2 diabetes risk is also known to be rooted in cultural, environmental, and behavioral factors that vary geographically, as reflected in the identification of a diabetes belt running across the Mississippi Valley, Deep South, and Appalachian region [14] and identification of counties with high diabetes rates clustering in the South and low diabetes counties clustering in West, Midwest, and Northeast [15]. Compared with the rest of the US, people in the diabetes belt and high prevalence cluster counties were more likely to be non-Hispanic African-American, lead a sedentary lifestyle, and be obese.

Despite the encouraging findings that diabetes incidence may be decreasing and that prevalence has plateaued, it is unknown whether such trends at the national level are apparent across all of the United States or whether improvements have been driven by selected geographic areas. We use publicly available county estimates to examine whether a slowing or leveling off in diagnosed diabetes, obesity, and leisure time physical inactivity prevalence is also evident across the 3143 counties of the US.

## Materials and methods

We assembled publicly available estimates of county level data on the prevalence of diagnosed diabetes (ever told by a health professional that they had diabetes), obesity (calculated from self-reported height and weight), and leisure time physical inactivity (no participation in any physical activities or exercises in the past month outside of regular job, henceforth referred to as physical inactivity) from 2004 to 2012 (http://www.cdc.gov/diabetes/atlas/countydata/County_ListofIndicators.html). These data were generated for all 3143 US counties by the Centers for Disease Control and Prevention (CDC) using Bayesian multilevel modeling techniques on 2004–2012 Behavioral Risk Factor Surveillance System (BRFSS) and U.S. Census data (http://www.cdc.gov/diabetes/atlas/countydata/County_ListofIndicators.html). The BRFSS is a CDC sponsored telephone survey that collects data in all 50 states, the District of Columbia, and three U.S. territories. Each year, more than 400,000 adults are interviewed about health-related risk behaviors, chronic health conditions, and use of preventive services. Included in the survey are questions designed to measure diabetes, obesity and physical inactivity. County-level estimates of diabetes, obesity, and physical inactivity for the over 3,100 counties or county equivalents (e.g., parish, borough, and municipality) in the United States were calculated based on indirect model-dependent estimates using Bayesian multilevel modeling techniques and were made publically available. The methods used to produce these county-level estimates have been previously described [16].

We contrasted changes in county level prevalences of diagnosed diabetes, obesity, and physical inactivity from 2004 to 2008 to the changes from 2008 to 2012. These 4 year time periods were selected because they coincided with trend changes in national estimates of diabetes prevalence [1]. Using multilevel Bayesian methods and cubic splines, we smoothed the publically available county-level estimates. We then used the smoothed estimates to calculate the average annual percentage point change (APPC) within each 4 year time period. This was done by substracting the estimate for the first year from the estimate of the fourth year and dividing by 4. The Appendix in S1 Appendix provides a more detailed explanation of the methods.

To examine county level change, we created US maps of the county APPCs for diagnosed diabetes, obesity, and physical inactivity for the 2 time periods. The five classes in the maps were calculated from natural breaks based on the Jenks methods [17], except that the lowest class was forced less than 0 to highlight decreases. The Jenks method of classification tries to reduce the variance within classes and maximize the variance between classes. We also examined change in the distributions of county APPCs between the two time periods overall and by Census region.

## Results

**Fig 1:**
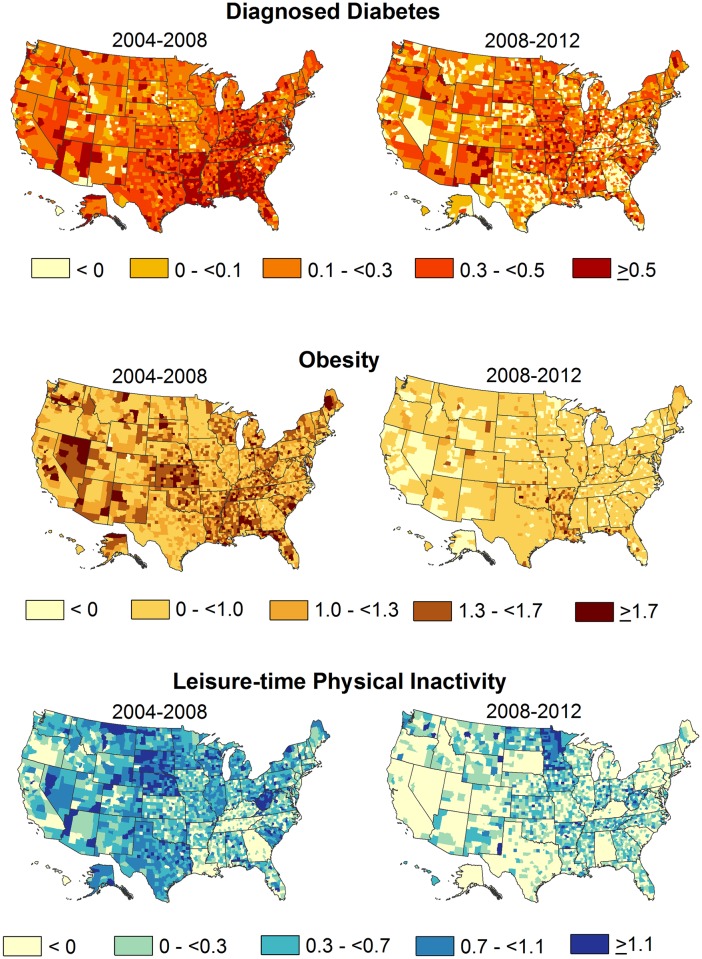
Average Annual Percentage Point Change (AAPC) in diagnosed diabetes, obesity, and physical inactivity prevalence, US counties, 2004–2008 and 2008–2012.

**Fig 2:**
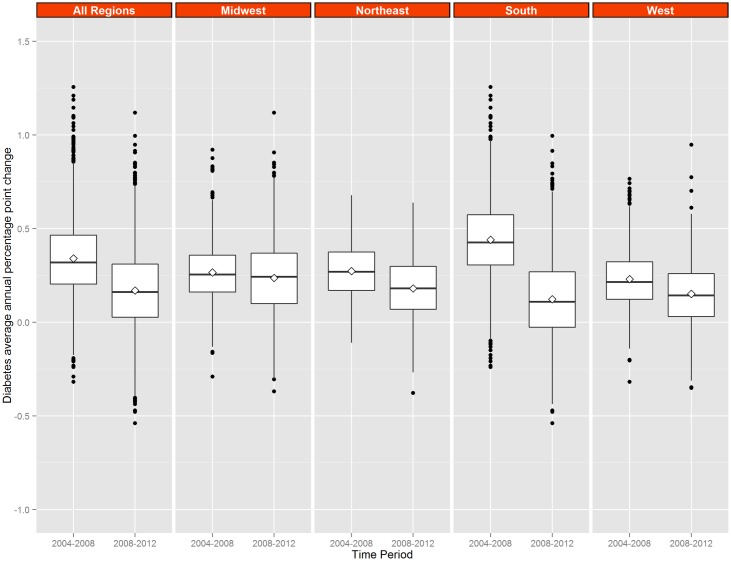
Distribution of county average Annual Percentage Point Change (APPC) in diagnosed diabetes, by Census region, 2004–2008 and 2008–2012. Northeast region includes Connecticut, Maine, Massachusetts, New Hampshire, New Jersey, New York, Rhode Island, Vermont, and Pennsylvania; Midwest region includes Illinois, Indiana, Iowa, Kansas, Michigan, Minnesota, Missouri, Nebraska, North Dakota, Ohio, South Dakota, and Wisconsin; South region includes Alabama, Arkansas, Delaware, District of Columbia, Florida, Georgia, Kentucky, Louisiana, Maryland, Mississippi, North Carolina, Oklahoma, South Carolina, Tennessee, Texas, Virginia, and West Virginia. The West region includes Arizona, Colorado, Idaho, Montana, Nevada, New Mexico, Utah, Wyoming, Alaska, California, Hawaii, Oregon, and Washington.

**Fig 3:**
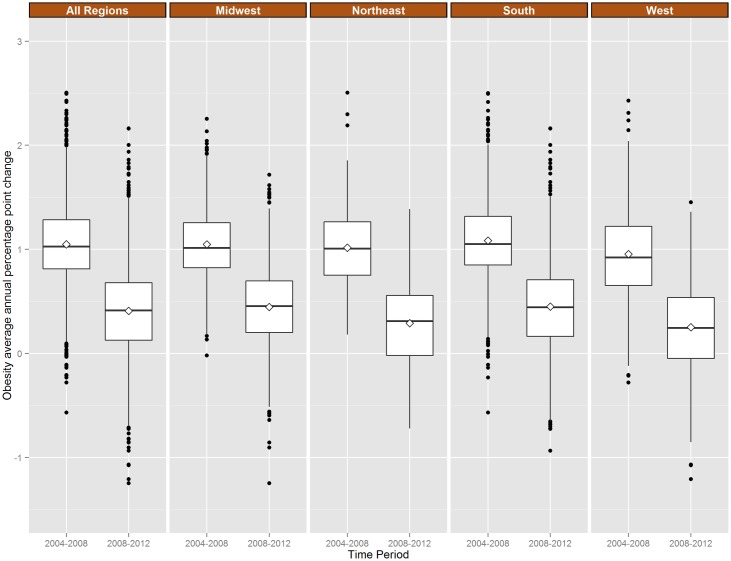
Distribution of county average Annual Percentage Point Change (APPC) in diagnosed obesity, by Census region, 2004–2008 and 2008–2012. Northeast region includes Connecticut, Maine, Massachusetts, New Hampshire, New Jersey, New York, Rhode Island, Vermont, and Pennsylvania; Midwest region includes Illinois, Indiana, Iowa, Kansas, Michigan, Minnesota, Missouri, Nebraska, North Dakota, Ohio, South Dakota, and Wisconsin; South region includes Alabama, Arkansas, Delaware, District of Columbia, Florida, Georgia, Kentucky, Louisiana, Maryland, Mississippi, North Carolina, Oklahoma, South Carolina, Tennessee, Texas, Virginia, and West Virginia. The West region includes Arizona, Colorado, Idaho, Montana, Nevada, New Mexico, Utah, Wyoming, Alaska, California, Hawaii, Oregon, and Washington.

**Fig 4:**
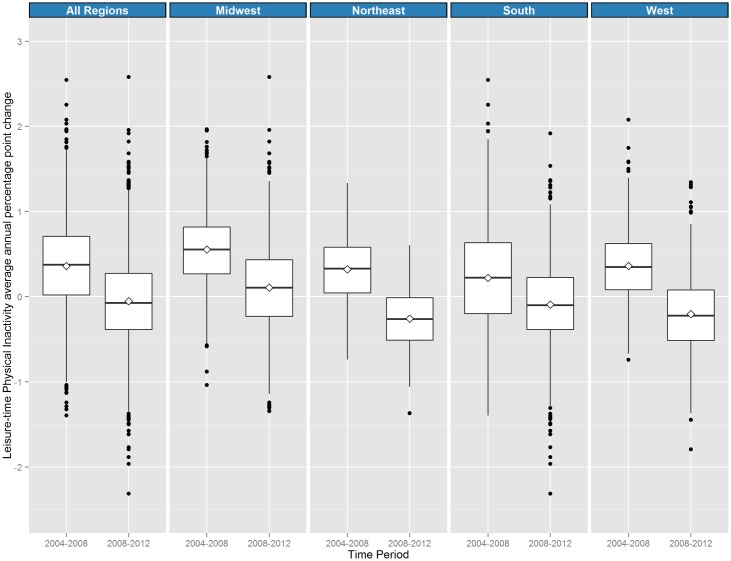
Distribution of county average Annual Percentage Point Change (APPC) in physical inactivity prevalence, by Census region, 2004–2008 and 2008–2012. Northeast region includes Connecticut, Maine, Massachusetts, New Hampshire, New Jersey, New York, Rhode Island, Vermont, and Pennsylvania; Midwest region includes Illinois, Indiana, Iowa, Kansas, Michigan, Minnesota, Missouri, Nebraska, North Dakota, Ohio, South Dakota, and Wisconsin; South region includes Alabama, Arkansas, Delaware, District of Columbia, Florida, Georgia, Kentucky, Louisiana, Maryland, Mississippi, North Carolina, Oklahoma, South Carolina, Tennessee, Texas, Virginia, and West Virginia. The West region includes Arizona, Colorado, Idaho, Montana, Nevada, New Mexico, Utah, Wyoming, Alaska, California, Hawaii, Oregon, and Washington.

US maps of county APPCs in diagnosed diabetes prevalence, obesity, and physical inactivity are displayed for both time periods in Fig 1a–1c. These county APPCs are further summarized by region (Figs 2–4). Counties with the largest increase in diagnosed diabetes between 2004 and 2008 (APPC ≥0.5, n = 649) were primarily located in Southern and Appalachian states, while most other counties having large increases were scattered throughout the West (Fig 1a). However, in 2008–2012, far fewer counties (n = 209) had APPCs ≥0.5 and many counties in the South now had APPCs <0.1. When the distributions of county APPCs are examined by region and time period, the South had the greatest downward shift in the distribution of county APPCs (Fig 2)(with the median APPC of 0.43 in 2004–2008 and 0.12 in 2008–2012, difference (d) = 0.31, 95% Bayesian posterior interval, 95%CI = 0.28, 0.35) and the Midwest had the smallest change (with a median APPC of 0.26 in 2004–2008 and 0.23 in 2008–2012, d = 0.03, 95%CI = 0.00, 0.07). The median APPC for all counties for 2004–2008 was 0.32 compared to 0.16 in 2008–2012 (d = 0.16, 95%CI = 0.14, 0.18). The correlation between county APPCs in the two time periods was r = -0.40 (95%CI = -0.43, -0.37).

In 2004–2008, 891 counties had APPCs ≥1.3 for obesity and these counties tended to be concentrated in Southern states (with the exception of Georgia and Texas) and in Wisconsin, Ohio, Oklahoma, Kansas and Nevada (Fig 1b). However, in 2008–2012, only 95 counties had APPCs ≥1.3 and these counties tended to cluster in Oklahoma, Arkansas, Louisiana, and the panhandle of Florida. Each regional distribution of county APPCs shifted downward and the overall US median APPC of 1.04 (95%CI = 1.01, 1.07) in 2004–2008 declined to 0.39 (95%CI = 0.36, 0.43) in 2008–2012 (d = 0.65, 95%CI = 0.59, 0.70) (Fig 3). There was negative correlation between county APPCs in obesity between the two time periods (r = -0.33, 95%CI = -0.36, -0.30). In both time periods, change in county obesity was correlated with change in diabetes prevalence (r = 0.76, 95%CI = 0.75, 0.77) in 2004–2008 and in 2008–2012 (r = 0.73, 95%CI = 0.72, 0.74).

For physical inactivity, counties with APPCs ≥1.1 (n = 246) in 2004–2008 appeared to be concentrated in West Virginia, Nebraska, South Dakota and scattered throughout the rest of the US (Fig 1c). In 2008–2012, far fewer counties (n = 74) had APPCs ≥1.1 and 1631 counties had negative APPCs. As with obesity, each regional distribution of physical inactivity APPCs shifted downward (Fig 4). In 2008–2012, the median county APPCs of the Northeast, South and West were negative (d = -0.26, 95%CI = -0.34, -0.18; d = -0.09, 95%CI = -0.14, -0.04; d = -0.22, 95%CI = -0.29, -0.15, respectively). Overall, the median APPC in 2004–2008 was 0.36 compared to -0.07 in 2008–2012 (d = 0.43, 95%CI = 0.37, 0.48). There was a negative correlation between 2004–2008 and 2008–2012 APPCs in leisure time physical activity (r = -0.34, 95%CI = -0.37, -0.30). Change in county physical inactivity was positively correlated with change in diabetes prevalence in both 2004–2008 (r = 0.53, 95%CI = 0.51, 0.55) and 2008–2012 (r = 0.64, 95%CI = 0.63, 0.66).

## Discussion

Following steady increases in diabetes prevalence over recent decades, national surveillance data suggest a peaking and plateauing in the prevalence of diagnosed diabetes occurred [1, 2]. The findings from our ecological study using different data suggest a slowing in prevalence is now evident at the county level. Our study suggests that the increase in diabetes prevalence slowed in US counties, with the average annual percentage point increase in 2008–2012 at half of the increase in 2004–2008. Our study also suggests that there may be important geographic variation in these changes as greater improvements were observed in the South. The growth in diabetes decreased by ~75% in the South, an important and encouraging finding given the South’s historic disproportionate burden of diabetes and its position at the heart of the “diabetes belt” [14, 18, 19]. Diabetes prevalence in counties is associated with socioeconomic factors (such as poverty, education, and race/ethnicity) and diabetes risk factors (such as obesity and physical inactivity) [14, 19]. The overall slowing in county level diabetes prevalence is consistent with the findings of several recent studies suggesting that the incidence or prevalence of diabetes may be slowing or plateauing in the U.S. overall and in selected populations [1, 2, 20–22]. Thus, our study adds to the growing evidence that the epidemic of diabetes in the US is slowing.

We did not examine reasons for the potential slowing in diabetes prevalence among US counties but it could be influenced by several factors, including a slowing in the growth of obesity [4, 5], improvements in diet [9–13], increasing physical activity [6–8], and changes to the diagnostic criteria [23] for diabetes, which could have led to changes in identification and detection of diabetes cases. Our analysis of county-level data documents coinciding improvements in two major risk factors for diabetes: obesity and physical inactivity. Between the two time periods, the median of the average annual percentage increase in county obesity prevalence declined ~60% and the median change in county estimates of physical inactivity went from an increase to a small decrease. A prior study using the same county-level data to examine changes in diabetes prevalence between 2004 and 2012 found a positive association between diabetes prevalence and baseline rates of physical inactivity and obesity [15]. Also, a study [24] using different methods to estimate and examine trends in county-level prevalence of physical activity and obesity in the US in the 2000s had findings consistent with our study. That study and ours found declines in physical inactivity and increasing obesity among US counties across the time period. However, in addition, our study found that the growth in obesity prevalence slowed in 2008–2012. Our findings are consistent with prior studies of nationally representative data suggesting that the rate of increase in obesity slowed or leveled off in the US [4, 5] and the diet of Americans improved in the mid to late 2000s [9–13]. The improvements in diabetes, obesity, and physical inactivity trends at the county and national level may spell good news for future trends in chronic diseases. However, despite these improvements, levels of these risk factors remain high, and some have merely slowed rather than reversing, suggesting that all counties would likely benefit from reductions in these risk factors. Further, the recent increase in obesity [25] seen at the national level—but occurring after the study period of our data—questions whether these positive trends will continue.

To our knowledge, the CDC county-level estimates used in our study are the only freely available annual estimates of diabetes, obesity, and physical inactivity with sufficient longevity to examine trends. Although CDC’s county estimates have been used in public health research to examine their cross-sectional associations with behavioral risk factors and physical and social environments and [26–29], the availability of several years of county estimates allowed our examination of county trends and also will allow more powerful examinations of how temporal changes in these variables are associated with physical and social environmental changes.

Despite the strengths of our data, there are several limitations to our study. CDC’s county estimates are in part based on the BRFSS, a state-based, telephone health survey and self-reported data have some limitations. Diabetes prevalence excludes persons with undiagnosed diabetes; obesity prevalence tends to be under estimated due to underestimates of body weight and overestimates of height [30]. Further, assessment of physical inactivity was based on responses to one question. Also, BRFSS data cannot distinguish between type 1 and type 2 diabetes. Because type 2 diabetes accounts for about 95% of all diabetes [31], our findings are likely more representative of type 2 diabetes. During most of the period studied, BRFSS was conducted using landline phones; cell phones were included starting in 2011. Depending on the impact of this survey change on prevalence estimates in the second time period, this could have led to either lower or higher estimates of APPCs than if the change had not occurred. Although we found no departure in trend in medians of county prevalence in 2011 [15], we cannot completely discount any impact of including cell phone respondents and it is also conceivable that any impact may not have been uniform over all counties. Last, the county estimates used here are model-based estimates and therefore are subject to the assumptions of these models.

## Conclusions

Our county level estimates show improvements in diabetes, obesity, and physical inactivity trends between 2004 and 2012 which are consistent with national trends during that time period. These estimates highlight geographic patterns of change in the prevalence of diabetes, obesity, and physical inactivity. Along with county prevalence estimates of these risk factors, awareness of how these factors are changing might assist local policy makers in targeting and tracking the impact of efforts to reduce these risk factors.

## Supporting information

S1 Appendix Detailed methods. (DOCX)
