# Supplementary material for: Changes in diagnosed diabetes, obesity, and physical inactivity prevalence in US counties, 2004-2012
Source: PLoS One. 2017 Mar 7;12(3):e0173428. doi: 10.1371/journal.pone.0173428 (PMC5340361; doi:10.1371/journal.pone.0173428)
Supplement: S1 Appendix — (DOCX) [file pone.0173428.s001.docx]

**S1 Appendix. Detailed methods**

To estimate the average annual percentage point changes (APPCs), a Bayesian multilevel model was fitted to the county-level estimates accounting for estimated variances. Our approach is similar to methods used in meta-analysis (Hedges and Olkin, 1985). We used cubic splines with knots at times (1, 4, 9) i.e., (2004, 2008, and 2012) to smooth the estimates.

Let the y_ij_, the estimated prevalence of diabetes in county i (i = 1, …, 3143) at time j (j = 1, …, 9, represents year 2004 through 2012) and the s_ij_^2^ are estimated variance of y_ij,._ These estimates are available from the CDC (<http://www.cdc.gov/diabetes/data/county.html>). We use cubic splines with knots at times (1, 4, 9) to smooth the estimates. Let *x_jk_* = the cubic spline matrix for time *j* (*j* = 1, …, 9) and knots *k* (*k* = 1, 2, 3). Let *s*(*i*) (*s*(*i*) = 1, …, 51) denote the state*s* that contains county *i*. Then,

$y_{ij} \sim\text{Normal}(\mu_{ij},s_{ij}^{2})$,

where, μ_ij_ is the mean for county i at time j and sij^2^ are assumed known.

The regression model was

$$\mu_{ij}= \sum_{k=1}^{3} \alpha_{k}x_{jk}+ \sum_{k=1}^{3} \beta_{ik}x_{jk}+ \sum_{k=1}^{3} \gamma_{s\left( i \right)k}x_{jk}+ \varepsilon_{ij}$$

where, $\alpha_{k}$ are fixed effects by cubic spline of time; $\beta_{ik}$ are random effects by county and cubic spline of time; $\gamma_{s\left( i \right)k}$ are random effects by state and cubic spline of time; $\varepsilon_{ij}$ are random error terms by county and time. For prior distribution, the fixed effects were assigned diffuse normal priors, $\alpha_{k} \sim\text{Normal (0, 10000).}$ The random effects by county and cubic spline of time, *β*, were assigned a multivariate (of dimension 3) normal prior with mean zero and variance matrix $\Sigma_{\beta}$. The inverse of $\Sigma_{\beta}$ was assigned a Wishart prior with scale matrix *S* and 3 degrees of freedom. The matrix *S* has ones along the main diagonal and 0.001 for all other elements (Rao, 2003). The random effects by state and cubic spline of time, *γ*, were assigned the same type of prior as *β*. The random error terms by county and time, *ε*, were assigned a normal prior with mean zero and variance $\tau^{-1}$ and $\tau\sim\text{Gamma}\left( 0.001, 0.001 \right).$

Let $\delta_{ik}= \alpha_{k}+\beta_{ik}+\gamma_{s\left( i \right)k}\text{ for }i=1, \ldots, 3143\text{ and} k=1, 2, 3.$

Then $\delta_{ik}$represents a smoothed value of prevalence for county $i\text{,}$ year 2004 (*k*=1), 2008 (*k*=2*)*, and 2012 (*k*=3).

The AAPC in smooth value of prevalence for i^th^ county between 2004 and 2008 was obtained as

$$(\delta_{i2}-\delta_{i1})/4$$

and between 2008 and 2012, obtained as

$(\delta_{i3}-\delta_{i2})/4$.

The means of the posterior distributions were used for point estimates of AAPC and the 95% highest posterior density intervals were used for confidence intervals. Models were fit using WinBUGS.

**References**

Hedges, L.V. and Olkin, I. (1985). *Statistical Methods for Meta-Analysis*, Chapter 9 – Random Effects Models for Effect Sizes. Pages 189–203 San Diego: Academic Press, Inc.
